# Proximity to Screening Site, Rurality, and Neighborhood Disadvantage: Treatment Status among Individuals with Sexually Transmitted Infections in Yakima County, Washington

**DOI:** 10.3390/ijerph17082679

**Published:** 2020-04-14

**Authors:** Solmaz Amiri, Christine D. Pham, Ofer Amram, Karl C. Alcover, Oladunni Oluwoye, Lilian Bravo, Melissa Sixberry, Michael G. McDonell, John M Roll, Andre Fresco

**Affiliations:** 1 Department of Nutrition and Exercise Physiology, Elson S. Floyd College of Medicine, Washington State University, Spokane, WA 99202, USA; ofer.amram@wsu.edu; 2 School of Molecular Biosciences, Washington State University, Pullman, WA 99163, USA; christine.d.pham@wsu.edu; 3 Program of Excellence in Addiction Research, Elson S Floyd College of Medicine, Washington State University, Spokane, WA 99202, USA; k.alcover@wsu.edu (K.C.A.); oladunni.oluwoye@wsu.edu (O.O.); mmcdonell@wsu.edu (M.G.M.); johnroll@wsu.edu (J.M.R.); 4 Yakima County Health District, Yakima, WA 98903, USA; Lilian.Bravo@co.yakima.wa.us (L.B.); melissa.sixberry@co.yakima.wa.us (M.S.); andref@co.yakima.wa.us (A.F.)

**Keywords:** GIS, Yakima County, sexually transmitted infections, treatment, proximity to testing site

## Abstract

*Background*: Early sexually transmitted infections (STIs) diagnosis facilitates prompt treatment initiation and contributes to reduced transmission. This study examined the extent to which contextual characteristics such as proximity to screening site, rurality, and neighborhood disadvantage along with demographic variables, may influence treatment seeking behavior among individuals with STIs (i.e., chlamydia, gonorrhea, and syphilis). *Methods*: Data on 16,075 diagnosed cases of STIs between 2007 and 2018 in Yakima County were obtained from the Washington State Department of Health Database Surveillance System. Multilevel models were applied to explore the associations between contextual and demographic characteristics and two outcomes: (a) not receiving treatment and (b) the number of days to receiving treatment. *Results:* Contextual risk factors for not receiving treatment or having increased number of days to treatment were living ≥10 miles from the screening site and living in micropolitan, small towns, or rural areas. Older age was a protective factor and being female was a risk for both outcomes. *Conclusions:* Healthcare providers and facilities should be made aware of demographic and contextual characteristics that can impact treatment seeking behavior among individuals with STIs, especially among youth, females, and rural residents.

## 1. Introduction

Over two million people annually in the United States are diagnosed with sexually transmitted infections (STIs) including chlamydia, gonorrhea, and syphilis [1]. In 2018, the United States rates of chlamydia, gonorrhea, and syphilis were 539.9, 179.1, and 10.8 per 100,000 population, respectively [1]. In the same year, Yakima County rates of chlamydia were 702.2, while Washington State had a rate of 467.9 per 100,000 population [2]. Rates of gonorrhea in Yakima County were 218.5 compared to 151.0 per 100,000 population in Washington State and syphilis rates were 19.6 in Yakima County compared to the Washington State rate of 10.9 per 100,00 population [2]. STI rates in Yakima County have been consistently higher than the state and national rates in the last decade [2].

The asymptomatic nature of STIs makes these infections a prevalent but hard to manage public health issue [3,4]. According to Centers for Disease Control and Prevention estimates, every new 20 million STIs impose approximately $16 billion in direct medical costs on the American healthcare system [5]. If left untreated, severe health consequences of STIs may include cancer, ectopic pregnancy, and infertility in women [4]. Men can suffer from urethritis, discharge from the urethra, and pain in the testicles [6].

Lack of healthcare infrastructure has contributed to the rise of STIs, particularly the closing of sites where underserved persons typically seek treatment [7,8]. Studies have explored the impact of proximity to healthcare services on health access and utilization [9]. In Wake County, North Carolina, gaps in the availability of services were a barrier to obtaining STI screening [10]. In rural Kenya, adolescent girls cited proximity to screening sites to be desirable, but preferred some distance between school and the screening facilities [11]. In France, increased distance to providers was negatively correlated with hepatitis C detection [12]. In Los Angeles County, low socio-economic individuals were less likely to use publicly funded medical clinics to get tested for human immunodeficiency virus (HIV) if they were located at farther distances [13].

Gaps in access to care are pronounced in rural areas [14]. Residents of rural communities tend to travel two to three times farther to seek medical care compared to their urban counterparts [14] and have been shown to have lower rates of STI screening [10,15,16]. A study of women enrolled in Medicaid in Pennsylvania showed that STI screening was lower among rural residents [15]. Analysis of the 2015 Behavioral Risk Factor Surveillance System data showed that rural residents were less likely to have ever or in the last year received a test for human immunodeficiency virus compared to urban residents [16]. Individuals living in rural areas face additional barriers such as stigma and perception of judgement by providers or their peers when it comes to being screened or tested for STIs [11,17].

Socioeconomic disparities contribute to the prevalence of STIs. Increased individual- and neighborhood-level poverty and income inequality are associated with higher rates of STIs [18,19]. Analysis of the National Longitudinal Study of Adolescent Health data showed a positive association between neighborhood poverty and the likelihood of being diagnosed with chlamydia [20]. In Pennsylvania, adolescents and young adults residing in areas with the highest poverty rates had higher rates of STIs compared to those living in other neighborhoods [19].

To our knowledge, the role of proximity to screening site, rurality, and neighborhood disadvantage on treatment seeking behavior among individuals with STIs is yet to be evaluated. The purpose of this study was to examine (a) whether the likelihood of not receiving treatment varied by proximity to screening sites, socio-economic context of the area of residence, and rurality; and (b) whether the number of days to treatment varied by proximity to screening sites, socio-economic context of the area of residence, and rurality among those who were treated for STIs.

## 2. Material and Methods

### 2.1. Study Area

Yakima County is located in south central Washington State with a population of 251,446 people and a total area of 4295 square miles [21]. Approximately 50% of the population of Yakima County are Hispanic or Latinos (compared to 13% in Washington State) and 7% are American Indian or Alaska Native (compared to 2% in Washington State). About one-fifth of land area of Yakima County is devoted to agricultural production and this figure does not include the vast farmland within the Yakama Indian Nation’s tribal trust lands. Median household income in this county is $47,470, which is lower than the median household income of $66,174 in Washington State. Approximately 18% of the population of Yakima County live in poverty, while that number stands near 10% in Washington State [21].

### 2.2. Study Design and Participants

Data for this study were obtained from the Washington State Department of Health Database Surveillance System. This dataset included diagnosed cases of chlamydia, gonorrhea, and syphilis with positive laboratory test results from January of 2007 through December of 2018. The dataset included information on the patient’s age, gender, race, ethnicity, home address, screening site name, test date, and treatment date if the individual received treatment. Of the total 19,800 cases from 2007 to 2018, we identified 16,075 (77%) cases with a valid geocodable home and screening site address. Our use of the data with indirect identifiers was considered exempt by the Washington State Institutional Board.

### 2.3. Measures

#### 2.3.1. Outcome Variables

We created two variables, (a) treatment status and (b) the number of days to treatment, to quantify treatment seeking behavior among individuals with STIs. Our outcomes of interest are described below.

*Treatment status* (binary). Our primary outcome was treatment status, which we defined as having received treatment for any of the STIs. The surveillance data included date of treatment if individuals received treatment for STIs. We used this data to create a binary variable and distinguish between individuals who received treatment and those who did not. Here, not receiving treatment was the outcome of interest.

*Number of days to treatment* (count). Our secondary outcome was the number of days to treatment measured by the timing between screening/diagnosis and treatment dates. The surveillance data included date of screening, diagnosis, and treatment. These data were used to calculate the number of days to treatment by subtracting the treatment date from the screening/diagnosis date for patients who received treatment only.

#### 2.3.2. Explanatory Variables

The explanatory variables included demographic and contextual characteristics. Demographic characteristics were described by age (continuous), gender (two categories: female, male), race (three categories: white, non-white, unknown), and ethnicity (three categories: Hispanic, non-Hispanic, unknown). Contextual characteristics were described by distance to the corresponding screening site, area deprivation index, and rural–urban commuting area codes.

*Distance to screening site* (categorical). The primary explanatory variable was distance to the corresponding screening site measured by the number of miles traveled from the nearest street intersection point from a patient’s self-reported residence to the screening site. Address points were geocoded to the nearest street intersection point to protect patient privacy. The addresses of screening sites were looked up using Google. The Environmental Systems Research Institute (ESRI) ArcGIS Business Analyst USA Local Composite geocoder and R software were used for geocoding the addresses. ESRI ArcGIS Network Analyst was utilized to calculate the number of miles between the latitude and longitude coordinates of the street intersection points and the latitude and longitude of the screening sites. Distance was calculated using the road network dataset of North America available in ArcGIS Business Analyst. Proximity was categorized as: (a) 5 miles or less, (b) between 5 and 10 miles, and (c) 10 miles or more.

*The Rural–Urban Commuting Area* (binary). The Rural–Urban Commuting Area (RUCA) codes will be used for delineating between urban and rural commuting areas [22,23]. RUCA codes utilize work commuting information, population data, and measures of urbanization to classify urban and rural areas. RUCA primary codes of 1–3 were classified as metropolitan areas, and RUCA primary codes of 4–10 included micropolitan, small town, and rural areas. RUCA codes were assigned to each individual based on their place of residence.

*Area deprivation index* (continuous). The area deprivation index (ADI) is a validated area-level disadvantage in the United States [24,25]. The ADI was developed by the University of Wisconsin School of Medicine and Public Health utilizing American Community Survey 5-year estimates at the block group level. This index included 17 census measures in the four domains of poverty, housing, employment, and education. The ADI is provided in the state percentile rankings from 1 to 10 with higher scores indicating greater deprivation.

### 2.4. Statistical Analysis

Univariate analyses included descriptive statistics with measures of central tendency and variability for continuous variables and frequency distributions and percentages for categorical variables. Unadjusted and adjusted multilevel binary logistic regression models were applied to explore the associations between not receiving treatment and contextual and demographic characteristics. Among those individuals who received treatment, multilevel negative binomial with log link models were applied to explore associations between the number of days to treatment and contextual and demographic characteristics. Individuals with STIs at level-1 were nested within census block groups at level-2. Application of multilevel modeling enabled the exploration of the effect of both individual- and area-level variables on the individual’s treatment seeking behavior while accommodating the clustering of individuals within block groups. Inclusion of random intercept in the multilevel models accommodated the likelihood of treatment seeking behavior to vary across block groups. Associations were presented as incidence odds ratios (ORs) with 95% confidence intervals (CIs). The data were analyzed using R. The significance level was set at 0.05 (two-tailed).

## 3. Results

**Figure 1:**
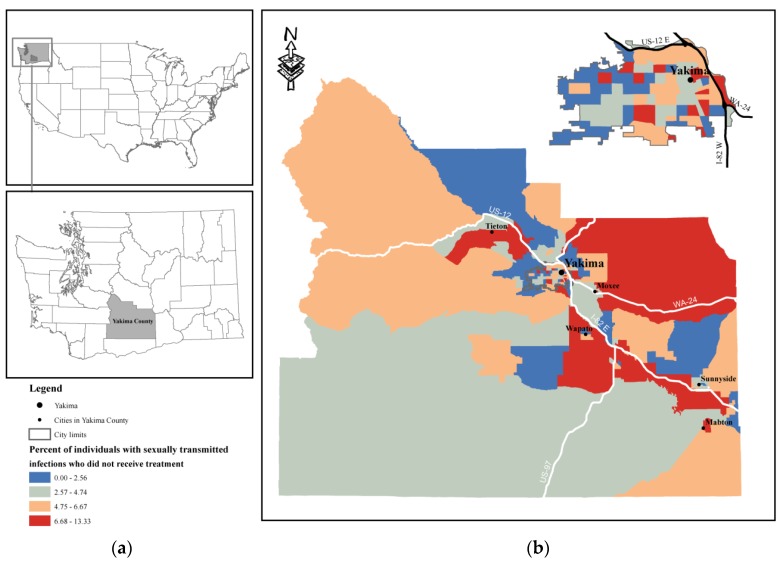
Study area location in the United States and Washington State (**a**) and STI rates in block groups in Yakima County and Yakima City (**b**).

The study sample included 16,075 patients who were diagnosed with STIs between 2007 and 2018. The sample’s mean age was 25 ± 8 years, 11,635 (72%) were female, 7435 (46%) were white, 8575 (53%) were Hispanics, and 12,700 (79%) lived in metropolitan areas. The average distance to testing sites was 10 ± 24.4 miles with an interquartile range of 1.6 to 8.2 miles. Approximately 95% percent of individuals who were screened and confirmed to have STIs received treatment. Figure 1 shows the study area and percentage of individuals with STIs who did not receive treatment. Individuals who did not receive treatment commuted 18 ± 33.2 miles on average to screening sites while those who received treatment traveled 9.8 ± 23.8 miles. The median number of days until treatment was two days with an interquartile range of 1–5 days. Patient characteristics are shown in Table 1.

*Treatment status.*Table 2 shows crude and adjusted ORs for the likelihood of not receiving treatment. Contextual risk factors for not receiving treatment were living ≥10 miles from the screening site (OR = 1.27, 95% CI = 1.05–1.53, *p* = 0.01) and living in micropolitan, small towns, or rural areas (OR = 1.40, 95% CI = 1.13–1.72, *p* = 0.002). Among the demographic characteristics, being female (OR = 1.27, 95% CI = 1.07–1.51, *p* = 0.01) was associated with increased risk of not receiving treatment. Older age was a protective factor against not receiving treatment (OR = 0.99, 95% CI = 0.98–0.99, *p* = 0.03).

In the adjusted model, risk factors for not receiving treatment were living ≥10 miles from the screening site (OR = 1.27, 95% CI = 1.05–1.53, *p* = 0.02), living in micropolitan, small towns, or rural areas (OR = 1.49, 95% CI = 1.20–1.86, *p* < 0.001), and living in successively more deprived areas (OR = 1.08, 95% CI = 1.01–1.16, *p* = 0.02). Among the demographic characteristics, being female (OR = 1.25, 95% CI = 1.05–1.50, *p* = 0.01) was associated with increased risk of not receiving treatment. Protective factor against not receiving treatment were older age (OR = 0.99, 95% CI = 0.98–0.99, *p* = 0.04) and being Hispanic (OR = 0.77, 95% CI = 0.63–0.93, *p* = 0.01).

*Number of days to treatment.*Table 3 shows the crude and adjusted ORs for increased number of days to treatment. Risk factors for delaying the treatment were living ≥10 miles from the screening site (OR = 1.10, 95% CI = 1.05–1.16, *p* < 0.001), living in micropolitan, small towns, or rural areas (OR = 1.33, 95% CI = 1.23–1.43, *p* < 0.001), and living in successively more deprived areas (OR = 1.03, 95% CI = 1.01–1.06, p = 0.002). Among the demographic characteristics, females (OR = 1.42, 95% CI = 1.36–1.47, *p* < 0.001) and Hispanics (OR = 1.12, 95% CI = 1.08–1.17, *p* < 0.001) were more likely to have increased number of days to treatment. Older age was a protective factor against increased number of days to treatment (OR = 0.99, 95% CI = 0.99–0.99, *p* < 0.001).

In the adjusted model, risk factors for delaying the treatment were living ≥10 miles from the screening site (OR = 1.13, 95% CI = 1.07–1.18, *p* < 0.001) and living in micropolitan, small towns, or rural areas (OR = 1.23, 95% CI = 1.14–1.33, *p* < 0.001). Among the demographic characteristics, females (OR = 1.38, 95% CI = 1.32–1.43, *p* < 0.001), non-whites (OR = 1.10, 95% CI = 1.06–1.15, *p* < 0.001), and Hispanics (OR = 1.12, 95% CI = 1.06–1.11, *p* < 0.001) were more likely to have increased number of days to treatment. Older age was a protective factor against increased number of days to treatment (OR = 0.99, 95% CI = 0.99–0.99, *p* < 0.001).

## 4. Discussion

The United States has experienced a steep and sustained increase in rates of STIs since 2014. STIs affect all populations, regardless of race, ethnicity, socioeconomic status, and religion [26,27,28]. Populations such as women, young people, and certain racial and ethnic categories (i.e., American Indian/Alaska Natives, African Americans, and Hispanics) are disproportionately affected [26,27,28]. Poverty, limited access to health care, substance use, and stigma associated with STIs also contribute to the prevalence of STIs [26,27,28].

In our study sample, 95% percent of individuals who were screened and confirmed to have STIs received treatment. The odds of not receiving treatment or having increased number of days to treatment was higher among patients who traveled ≥10 miles from their residence to the screening site compared to those who traveled ≤5 miles. Patients who lived ≤5 miles or between 5 and 10 miles from the screening site did not differ in terms of our outcomes of interest. While these findings are unique to this study, prior research found that access to care is essential for behavior-change counseling and screening, diagnosis, and treatment of STIs [28]. Living in micropolitan, small towns, or rural areas increased the odds of not receiving treatment or having increased number of days to treatment compared to those living in metropolitan areas. This finding is in line with previous studies highlighting lower rates of STI screening among rural residents [15,16] and the fact that rural residents may have to travel two to three times farther to seek medical care compared to their urban counterparts [14]. These findings suggest the need to improve spatial availability and accessibility of screening sites to improve treatment outcomes among individuals with STIs. Incorporating rapid testing for STIs so that test results are available on the same day for individuals who may have difficulties commuting back and those who may more likely be tested positive are warranted.

Higher area-level deprivation was associated with increased risk of not receiving treatment. This is similar to previous research showing higher diagnosed cases of STIs with increased neighborhood-level poverty [18,19]. Educational material, workshops, and resources should be made freely available in neighborhoods of lower socioeconomic status to provide information on safe sexual behavior, the importance of screening for STIs, and adverse outcomes associated with untreated STIs. Such resources can lead to reductions in the transmission of STIs, the duration of infection, the development of complications, and the health and economic burden of STIs [29,30]. Healthcare providers should be not only reeducated or refreshed on re-testing and screening practices, but also made aware of contextual characteristics that can influence people’s treatment seeking behavior.

Older age was a protective factor against not receiving treatment or having increased number of days to treatment. It is likely that older individuals are more aware of the importance of reproductive health screening and treatment. Youth and young adults, especially under the age of 25 years, are considered a high risk population for STIs [31]. Health education and screening efforts should be focused on this population who are sexually active including facilitation of human papillomavirus vaccine uptake [32]. Females were more likely to leave untreated or delay the treatment initiation compared to males. Research has shown that females tend to underutilize reproductive health screening and are disproportionately affected by STIs [26,27,28,33]. It may be the case that females bear the stigma associated with STIs, while males seek treatment for such infections. Approximately 10–15% of untreated cases among females may lead to severe health outcomes such as pelvic inflammatory disease [31]. Thus, understanding factors that can motivate or facilitate females to seek STI screening and treatment is critical. Providers should be aware of such disparities so that they can provide appropriate care according to the needs and characteristics of individuals, specifically youth, young adults, and females.

Hispanic or Latinos constitute approximately 50% of the population of Yakima County. Hispanics are disproportionately affected by STIs [26,27,28]. This ethnic population were less likely to leave untreated, but had increased number of days to treatment. Hispanics are more likely to be screened for STIs than non-Hispanics [15], and travel shorter distances to primary care (here screening sites) compared to non-Hispanics [34]. Understanding factors associated with increased number of days to treatment among Hispanics and encouraging non-Hispanics to follow up on their test results and receive treatment are warranted. Race was not a predictor for not receiving treatment, while non-whites were more likely to have an increased number of days to treatment. Approximately 25% of individuals with STIs did not report a race and 18% did not report an ethnicity. Understanding to what extent not declaring a race or ethnicity can be related to treatment seeking behavior among this population are open avenues for future research. Culturally sensitive tools targeted at different ethnic and racial populations should be developed. It is not enough to simply have materials translated to languages other than English. Materials should accurately represent the cultural values of different population and be developed in a collaborative effort with these racial and ethnic groups. Timely treatment of partners of individuals with STIs is also critical to reduce the duration of infection and the development of complications [35].

This study’s strengths include geocoding the home and testing site locations for more than 16,000 individuals who were diagnosed with STIs during a 12-year period between 2007 and 2018. The surveillance data enabled the study team to examine the effect of contextual characteristics on the likelihood of not receiving treatment and increased number of days to treatment. There are also some limitations to be taken into consideration. We quantified proximity as the number of miles traveled between the patient’s residence and the corresponding screening site. Distance may not be a perfect representation for time taken to reach to those destinations. Future research could take into consideration the mode of transport to better capture how travel time may influence treatment seeking behavior of individuals with STIs.

Individuals who were screened did not necessarily choose the nearest screening site. We did not know what factors might have been associated with these decisions. Access to screening sites for some groups such as homeless population, individuals with substance use disorders, or incarcerated individuals may raise concerns around availability and accessibility of treatment for this population. The surveillance data included date of treatment reported by primary care providers if individuals received treatment for STIs. Reporting date of treatment does not necessarily mean that the planned treatment was completed. We did not have information on the incidence of treatment failure among those who received treatment. Due to the concern surrounding treatment options and antibiotic-resistant gonorrhea, a formal system should be implemented for healthcare providers to report treatment failures. Different practice environments may have different approaches for when and how test results are conveyed to patients. These differences can influence the timing of treatment initiation and the delays we observed in treatment initiation.

This study included diagnosed cases with positive laboratory test results, not individuals. Some people might have been diagnosed with a chlamydia, gonorrhea, and syphilis at different time points. We were not able to identify such cases because we did not have information on unique identifiers such as the individual’s social security number. This might impact the results of this study. However, the statistics generated by other agencies suffer from the same limitation. The sample studied here may not be representative of the individuals with STIs across the United States.

## 5. Conclusions

In conclusion, contextual risk factors for not receiving treatment or having increased number of days to treatment were living ≥10 miles from the screening site and living in micropolitan, small towns, or rural areas. Older age was protective and being female was a risk factor for not receiving treatment or having increased number of days to treatment. Findings suggest the need to improve availability and accessibility of STI services. Healthcare providers and facilities should also be made aware of demographic and contextual characteristics that can impact treatment seeking behavior among individuals with STIs so that they can provide appropriate care according to the needs and characteristics of individuals.

## Figures and Tables

**Table 1:** Characteristics of patients diagnosed with sexually transmitted infections in Yakima County, Washington between 2007 and 2018, overall and stratified by treatment status (n = 16,075).

Characteristics	Total (No. (%))	Treatment Received		Days Until Treatment *
		Yes (15,313, 95%)	No (762, 5%)	
Proximity				
≤5 Miles	9676 (60.19)	9244 (60.37)	432 (56.59)	4.19 (7.53)
5 < x < 10 Miles	3123 (19.43)	2981 (19.47)	142 (18.64)	4.48 (14.88)
≥10 Miles	3276 (20.38)	3088 (20.17)	188 (24.67)	4.90 (10.26)
RUCA				
Metropolitan	12,700 (79.00)	12,138 (79.27)	562 (73.75)	4.10 (7.37)
Micropolitan, Small town, or Rural	3375 (21.00)	3175 (20.73)	200 (26.25)	5.51 (16.32)
Area deprivation index *	9.11 (1.44)	9.11 (1.45)	9.20 (1.27)	9.11 (1.44)
Age *	24.87 (8.10)	24.90 (8.11)	24.24 (7.84)	24.87 (8.10)
Gender				
Male	4435 (27.59)	4260 (27.82)	175 (22.97)	3.43 (13.17)
Female	11,635 (72.38)	11,049 (72.15)	586 (76.90)	4.76 (8.33)
Unknown	5 (0.03)	4 (0.03)	1 (0.13)	3.50 (3.79)
Race				
White	7435 (46.25)	7080 (46.24)	355 (46.59)	4.31 (11.52)
Non-white	4702 (29.25)	4497 (29.37)	205 (26.90)	4.94 (9.99)
Unknown	3938 (24.50)	3736 (24.40)	202 (26.51)	3.89 (5.65)
Ethnicity				
Non-Hispanic	4575 (28.46)	4357 (28.45)	218 (28.61)	4.04 (7.17)
Hispanic	8575 (53.34)	8219 (53.67)	356 (46.72)	4.77 (11.84)
Unknown	2925 (18.20)	2737 (17.87)	188 (24.67)	3.8 (6.93)

Note: * Mean (Standard deviation).

**Table 2:** Unadjusted and adjusted multilevel binary logistic regression analyses of characteristics associated with not receiving treatment in Yakima County, Washington between 2007 and 2018.

Characteristics	Unadjusted		Adjusted	
	OR (95% CI)	*p*	OR (95% CI)	*p*
Proximity				
≤5 Miles	Reference		Reference	
5 < x < 10 Miles	1.01 (0.82–1.25)	0.91	1.00 (0.80–1.24)	0.99
≥10 Miles	1.27 (1.05–1.53)	0.01	1.27 (1.05–1.53)	0.02
RUCA				
Metropolitan	Reference		Reference	
Micropolitan, Small town, or Rural	1.40 (1.13–1.72)	0.002	1.49 (1.20–1.86)	<0.001
Area deprivation index	1.06 (0.99–1.13)	0.07	1.08 (1.01–1.16)	0.02
Age	0.99 (0.98–0.99)	0.03	0.99 (0.98–0.99)	0.04
Gender				
Male	Reference		Reference	
Female	1.27 (1.07–1.51)	0.01	1.25 (1.05–1.50)	0.01
Race				
White	Reference		Reference	
Non-white	0.88 (0.74–1.06)	0.18	0.85 (0.70–1.02)	0.08
Unknown	1.09 (0.91–1.30)	0.37	0.94 (0.77–1.15)	0.54
Ethnicity				
Non-Hispanic	Reference		Reference	
Hispanic	0.84 (0.70–1.00)	0.06	0.77 (0.63–0.93)	0.01
Unknown	1.38 (1.13–1.69)	0.002	1.41 (1.12–1.78)	0.003

**Table 3:** Unadjusted and adjusted multilevel negative binomial with log link analyses of characteristics associated with the number of days to treatment in Yakima County, Washington between 2007 and 2018.

Characteristics	Unadjusted		Adjusted	
	OR (95% CI)	*p*	OR (95% CI)	*p*
Proximity				
≤5 Miles	Reference		Reference	
5 < x < 10 Miles	0.99 (0.93–1.04)	0.68	1.00 (0.95–1.06)	0.89
≥10 Miles	1.10 (1.05–1.16)	<0.001	1.13 (1.07–1.18)	<0.001
RUCA				
Metropolitan	Reference		Reference	
Micropolitan, Small town, or Rural	1.33 (1.23–1.43)	<0.001	1.23 (1.14–1.33)	<0.001
Area deprivation index	1.03 (1.01–1.06)	0.002	1.01 (1.00–1.03)	0.14
Age	0.99 (0.99–0.99)	<0.001	0.99 (0.99–0.99)	<0.001
Gender				
Male	Reference		Reference	
Female	1.42 (1.36–1.47)	<0.001	1.38 (1.32–1.43)	<0.001
Race				
White	Reference		Reference	
Non-white	1.13 (1.08–1.18)	<0.001	1.10 (1.06–1.15)	<0.001
Unknown	0.91 (0.87–0.95)	<0.001	0.93 (0.88–0.97)	0.002
Ethnicity				
Non-Hispanic	Reference		Reference	
Hispanic	1.12 (1.08–1.17)	<0.001	1.11 (1.06–1.16)	<0.001
Unknown	0.93 (0.89–0.98)	0.01	0.99 (0.94–1.05)	0.85
